# Addition of Kidney Dysfunction Type to MELD-Na for the Prediction of Survival in Cirrhotic Patients Awaiting Liver Transplantation in Comparison with MELD 3.0 with Albumin

**DOI:** 10.3390/diagnostics14010039

**Published:** 2023-12-25

**Authors:** Kyeong-Min Yeom, Jong-In Chang, Jeong-Ju Yoo, Ji Eun Moon, Dong Hyun Sinn, Young Seok Kim, Sang Gyune Kim

**Affiliations:** 1 Department of Internal Medicine, SoonChunHyang University School of Medicine, Bucheon 14584, Republic of Korea; 121931@schmc.ac.kr (K.-M.Y.); liverkys@schmc.ac.kr (Y.S.K.); 2 Department of Medicine, Chung-Ang University Gwangmyeong Hospital, Gwangmyeong 06973, Republic of Korea; imjic26@naver.com; 3 Department of Statistics, SoonChunHyang University School of Medicine, Bucheon 31538, Republic of Korea; moon6188@schmc.ac.kr; 4 Department of Medicine, Samsung Medical Center, Sungkyunkwan University School of Medicine, Seoul 06351, Republic of Korea; sinndhn@hanmail.net

**Keywords:** liver transplant, allocation, MELD, prognosis

## Abstract

It is well known that renal dysfunction has a devastating effect on the prognosis of liver cirrhosis. In this study, the aim was to assess whether the incorporation of the kidney dysfunction type into the MELD-Na score enhances its predictive capacity for outcomes in patients awaiting liver transplantation (LT), compared to utilizing the MELD 3.0 score with albumin. In total, 2080 patients awaiting the LT were enrolled at two tertiary care institutions in Korea. Discrimination abilities were analyzed by using Harrell’s c-index and iAUC values between MELD-Na-kidney dysfunction type (MELD-Na-KT) and MELD 3.0 with albumin. Clinical endpoints encompassed 3-month survival, 3-month transplant-free survival (TFS), overall survival (OS), and total TFS. Out of the total of 2080 individuals, 669 (32.16%) were male. Regarding the types of renal function impairment, 1614 (77.6%) were in the normal group, 112 (5.38%) in the AKD group, 320 (15.35%) in the CKD group, and 34 (1.63%) were in the AKD on CKD group. MELD 3.0 with albumin showed better discrimination (c-index = 0.714) compared to MELD-Na-KT (c-index = 0.708) in predicting 3-month survival. Similar results were observed for OS, 3-month TFS, and total TFS as well. When divided by sex, MELD 3.0 with albumin showed the comparable prediction of 3-month survival to MELD-Na-KT (c-index 0.675 vs. 0.671, *p*-value 0.221) in males. However, in the female group, MELD 3.0 with albumin demonstrated better results compared to MELD-Na-KT (c-index 0.733 vs. 0.723, *p*-value 0.001). The integration of kidney dysfunction types into the MELD-Na did not yield superior prognostic results compared to the MELD 3.0 score with albumin. Rather, in the female group, the MELD 3.0 score with albumin was better able to predict survival. These findings suggest that laboratory values pertaining to liver dysfunction or creatinine levels may be more significant than the type of kidney dysfunction when predicting the short-term prognosis of LT candidates.

## 1. Introduction

Liver transplantation (LT) stands out as a promising and life-saving treatment, with a 5-year survival rate exceeding 70%. However, the growing severity of the donor shortage in recent times has brought to light the pressing need for an enhanced allocation system [1]. These allocation systems play a crucial role in systematically identifying patients with a poor prognosis without transplantation. It is noteworthy that these systems exhibit subtle variations across different countries, reflecting the unique healthcare landscapes and patient populations [2,3]. In light of the dynamic nature of liver diseases and their evolving etiology, there has been a noticeable shift in prognostic outcomes over time [4,5,6]. This emphasizes the importance of continually refining and updating the allocation systems to align with the changing landscape of liver diseases. A key consideration is the development of an allocation system that not only adapts to the changing nature of liver diseases but also accurately predicts prognosis, taking into account the diverse factors influencing patient outcomes [7].

The original MELD score, developed for the allocation system, is calculated based on serum creatinine, bilirubin, and INR. Subsequently, through ongoing research, the MELD-Na incorporating sodium levels has been developed, demonstrating improved prognostic prediction. However, recognizing the need to address sex-related imbalances and further refine the predictive accuracy, recent endeavors have led to the development of MELD 3.0. MELD 3.0 represents a significant advancement in the allocation scoring system by introducing additional factors such as albumin and a sex factor. MELD 3.0 is calculated by adding the albumin level and sex factor to the MELD-Na system. This is because the level of albumin is closely related to the severity of liver disease, and women, who generally have less muscle mass, were at a disadvantage in the previous scoring system based on creatinine. This update aims to address this issue.

The introduction of the MELD 3.0 scoring system has demonstrated superior performance compared to the conventional MELD scoring system [8,9]. Based on our recent study findings, which focused on observation in the Asian population, it appears that the performance of the MELD 3.0 scoring system may exhibit a slight lag when compared to Western populations [10,11].

The evaluation of kidney dysfunction in the prognosis of liver cirrhosis holds paramount importance [12,13,14,15,16]. This underscores why the MELD scoring system incorporates serum creatinine levels as a crucial factor [17,18]. Recent research has reported that not only the degree of kidney dysfunction but also the type of dysfunction can be of significance in predicting the prognosis [19,20,21]. In response, the MELD-Na-KT scoring system has been proposed, which integrates the kidney dysfunction type alongside the MELD-Na score [22]. Because the above scoring system was formulated using data from a Western cohort, we conducted this study to investigate and evaluate the prognostic impact within an Asian cohort. This endeavor aimed to determine the prognostic relevance of kidney dysfunction types in diverse populations.

## 2. Materials and Methods

### 2.1. Patients

We conducted a retrospective analysis using a cohort database of cirrhotic patients awaiting LT at two tertiary hospitals in South Korea (Samsung Medical Center and Soonchunhyang University Bucheon Hospital). The study period spanned from January 2017 to December 2019. Inclusion criteria required that patients met the following conditions: (a) being above 19 years of age, (b) presenting evidence of cirrhosis determined through either imaging or laboratory examinations, and (c) being registered on the waitlist of the Korean Network for Organ Sharing (KONOS) for a deceased donor LT. Patients with current or prior evidence of hepatocellular carcinoma were excluded. The final dataset comprised 2080 patients, whose clinical and laboratory records were retrospectively reviewed. The study’s research protocol was approved by the Institutional Review Boards (IRBs) of both hospitals where the data were gathered (IRB number: SCHBC 2022-01-020, registered on 16 February 2022). The study protocol conformed to the ethical guidelines of the World Medical Association Declaration of Helsinki.

### 2.2. Kidney Dysfunction Type Definitions

We defined kidney dysfunction types as follows.

- AKD: an increase in sCr by either 0.3 mg/dL, ≥50%, or <72 days of hemodialysis;
- CKD: an eGFR < 60 mL/min/1.73 m2 for 90 days or ≥72 days of hemodialysis;
- AKD on CKD: meeting both AKD and CKD definitions;
- Normal kidney function: meeting neither definition.

### 2.3. Evaluation of Liver Function

To evaluate liver function, we computed various MELD-based scores using specific formulas. We utilized the formula proposed in a recently published paper to calculate the MELD-Na-KT score. The calculation of MELD-based scores was performed at the time of patient enrollment in KONOS.

- MELD original = 9.57 × log_e_(creatinine) + 3.78 × log_e_(bilirubin) + 11.20 × log_e_(INR) + 6.43.
- MELD-Na = MELD original + [1.32 × (137-Na)] − [0.033 × MELD original × (137-Na)].
- MELD-Na-KT (10):
  - AKD: (MELD-Na × 0.035480 − 0.318060)/(0.134426 + 0.035480);
  - CKD: (MELD-Na × 0.038190 − 1.087994)/(0.134426 + 0.038190);
  - AKD on CKD: (MELD-Na × 0.036395 − 0.488997)/(0.134426 + 0.036395).
- MELD 3.0 with albumin = 1.33 (if female) + [4.56 × log_e_(bilirubin)] + [0.82 × (137-Na)] − [0.24 × (137-Na) × log_e_(bilirubin)] + [9.09 × log_e_(INR)] + [11.14 × log_e_(creatinine)] + [1.85 × (3.5-albumin)] − [1.83 × (3.5-albumin) × log_e_(creatinine)] + 6.

### 2.4. Outcomes

The primary objective of this study was to compare the effectiveness of two MELD-based scores in predicting 3-month survival in patients awaiting LT. The secondary objective of the study was to assess the efficacy of these scores in predicting 3-month transplant-free survival (TFS), overall survival (OS), and total TFS based. Transplant-free survival (TFS) was defined as the duration between the registration date for LT at KONOS and either the date of death or the date of transplantation. Overall survival (OS) was defined as the duration from the registration date to death from any cause. The registration date represents when the patients registered for an LT at KONOS.

### 2.5. Statistical Analysis

Two criteria were employed to assess the performance and validate the predictive accuracy of each model. The discrimination ability for the prediction was assessed by Harrell’s concordance index (c-index) and the integrated time-dependent area under the curve (Heagerty’s iAUC). The c-index and iAUC had better performance as they more closely approached 1.

Model comparisons were conducted by measuring the difference in c-index estimates, along with 95% confidence intervals, using bootstrapping with 200 resamples and a calculated *p*-value. If the 95% confidence interval (CI) included zero, it indicated no statistical difference between the models. To assess the consistency between predicted probabilities and actual probabilities, calibration curves were generated through bootstrapping with 200 resamples. The survcomp package in the R program was used to calculate *p*-values for model comparisons.

We also conducted a sensitivity analysis, including subgroup analyses for both male and female patients. A subgroup analysis for kidney dysfunction types was additionally conducted for further evaluation. A two-sided *p*-value less than 0.05 was considered statistically significant. All statistical analyses were performed using R version 3.6.3 (The R Foundation for Statistical Computing, Vienna, Austria) with the survivalROC, survival, risksetROC, boot, rms, and survcomp packages.

## 3. Results

### 3.1. Baseline Characteristics

Table 1 provides the fundamental attributes of the 2080 patients enrolled on the LT waitlist through the study period (2017–2019). The average age of the patients was 52.53 ± 10.95 years, while males accounted for 67.84%. Hepatitis B constituted the primary etiology of cirrhosis (39.42%), followed by alcohol-induced cirrhosis (8.61%). Overall, 75.77% of the patients had some degree of ascites, while 38.94% exhibited some degree of hepatic encephalopathy. The patients’ MELD scores showed slight variation depending on the calculation method: MELD 3.0 with albumin yielded 20.15 ± 9.66, and MELD-Na-KT resulted in 19.69 ± 10.09. The MELD 3.0 scores with albumin were slightly higher than those of MELD-Na-KT, although there was no statistical significance.

### 3.2. Performance Comparison between MELD-Na-KT and MELD 3.0

We evaluated the predictive performance of the two MELD-based score systems for 3-month survival, OS, 3-month TFS, and overall TFS. In our previous study, MELD 3.0 demonstrated superior predictive accuracy compared to the original MELD. Consequently, we selected MELD 3.0 as the comparator for MELD-Na-KT in this study. To validate the predictive efficacy of each scoring system, the discrimination ability was evaluated by both Harrell’s c-index and iAUC (Table 2). Both MELD-based scores showed the highest predictive power for 3-month TFS, followed by overall TFS and 3-month survival. However, the discrimination ability of the MELD-based scores was relatively lower when predicting OS compared to their performance in predicting 3-month survival or 3-month TFS. In the comparison between the two scoring systems, MELD 3.0 with albumin demonstrated better performance than MELD-Na-KT in all categories, including 3-month survival, overall survival, 3-month transplant-free survival (TFS), and overall TFS (Table 2).

**Figure 1:**
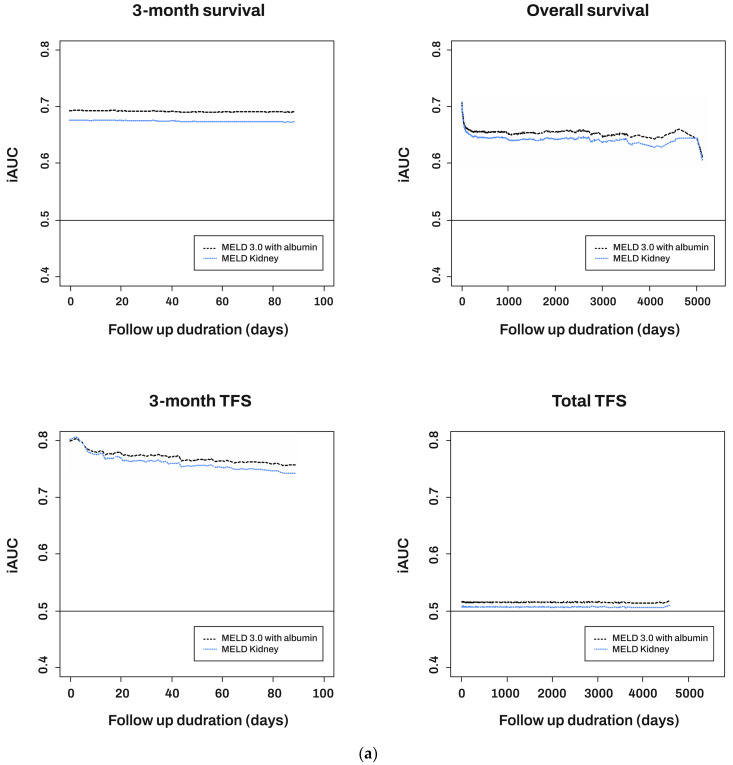

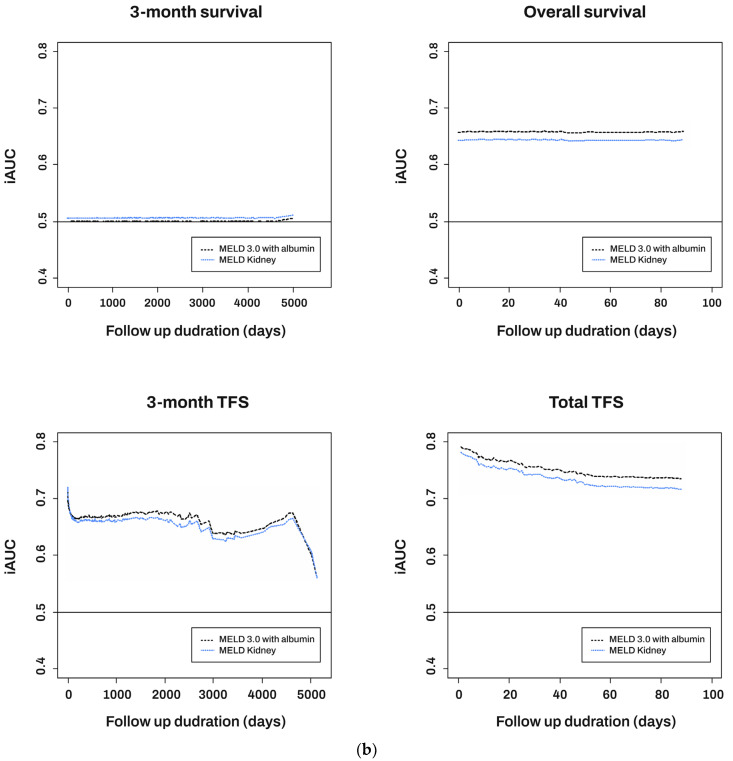

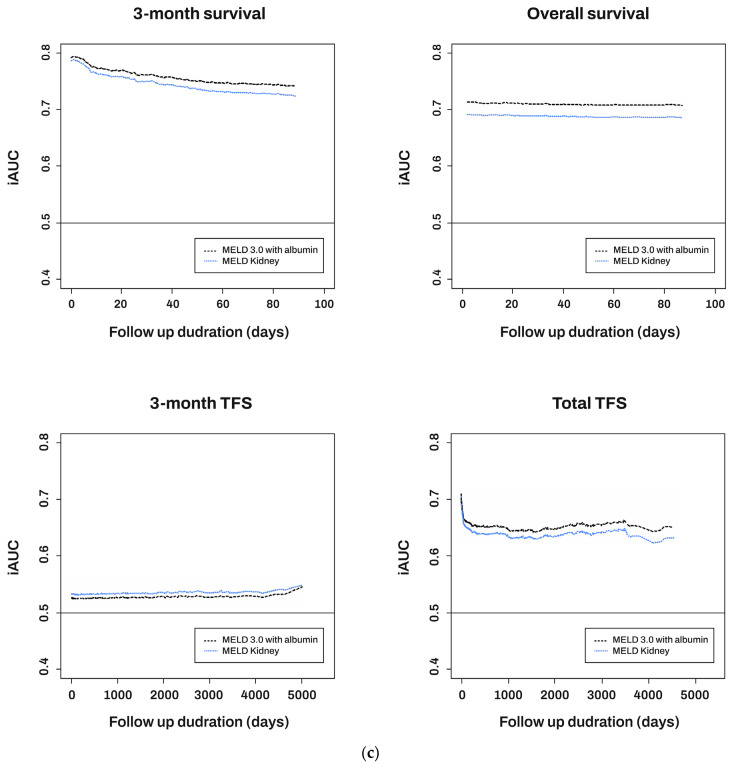
Discrimination ability by time-dependent receiver operating characteristic (ROC) analysis. (**a**) Total, (**b**) male, (**c**) female.

**Figure 2:**
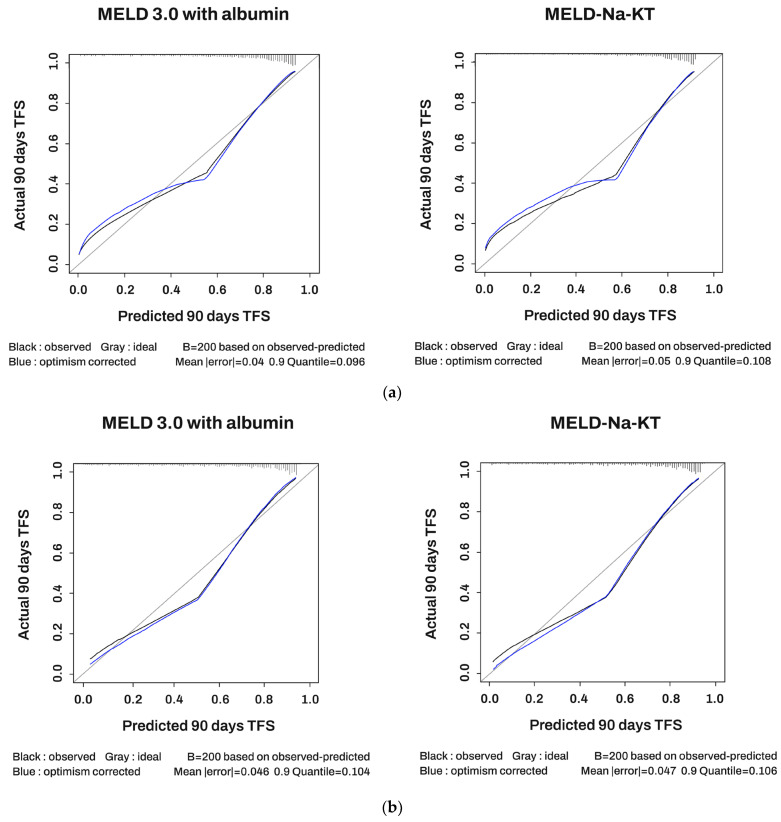

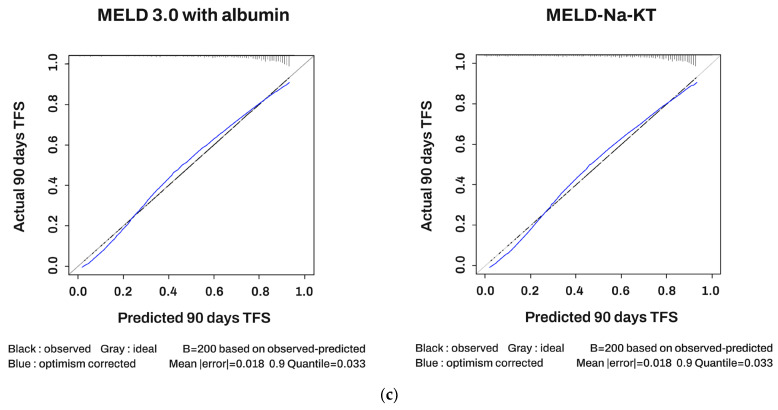
Calibration plot of 3-month transplant-free survival. (**a**) Total, (**b**) male, (**c**) female.

To examine the variation in discrimination ability over time, the MELD-based scores were compared using the time-dependent iAUC (Figure 1a). At all time points examined, the MELD 3.0 with albumin showed a higher iAUC value in predicting 3-month survival, TFS, and overall TFS compared to MELD-Na-KT. Regarding the prediction of overall survival, MELD-Na-KT demonstrated a higher iAUC value. Figure 2 shows a calibration plot for 3-month TFS. For both scoring systems based on the MELD, the predicted and actual probability values showed close correspondence.

### 3.3. Stratified Analysis According to Sex

The predictive efficacy between the two MELD-based scoring systems within subgroups was evaluated. When compared by sex, both MELD-based scores showed the highest predictive accuracy for 3-month TFS, followed by overall TFS, 3-month survival, and overall survival, in both males and females. In the comparison between the two scoring systems, MELD 3.0 with albumin demonstrated superior values to MELD-Na-KT in all categories, including 3-month survival, overall survival, 3-month transplant-free survival (TFS), and overall TFS, for males and females (Table 2). Nevertheless, the 3-month survival value for males did not have statistical significance in the comparison. In addition, we also conducted a comparison of the time-dependent iAUC by sex. In males, the MELD 3.0 with albumin showed a higher iAUC value when predicting 3-month survival, TFS, and overall TFS at all time points. However, MELD-Na-KT demonstrated a higher iAUC value when predicting overall survival. In females, the MELD 3.0 with albumin showed higher iAUC values in all categories.

### 3.4. Stratified Analysis According to Kidney Dysfunction Type

Next, we analyzed whether there was a difference in predictive efficacy between MELD 3.0 and MELD-Na-KT based on the type of kidney dysfunction (Table 3). Both MELD-based scores had the highest predictive accuracy for 3-month TFS, followed by overall TFS, 3-month survival, and overall survival, in all kidney dysfunction types. In the comparison between the two scoring systems, MELD 3.0 with albumin demonstrated superior performance over MELD-Na-KT in 3-month survival and overall survival across all kidney dysfunction types. However, the compared values for overall survival in the AKD and CKD groups did not reach statistical significance. The compared value for 3-month TFS and overall TFS in the normal, AKD, and AKD on CKD groups did not show statistical significance. Only values compared in the CKD group showed significance, and MELD 3.0 with albumin had higher predictive power than MELD-Na-KT. We conducted a comparison of the time-dependent iAUC for the two MELD scoring systems based on the kidney dysfunction type. First, in the group with normal kidney function, MELD 3.0 with albumin showed a higher iAUC value in 3-month and overall survival, while showing similar values for 3-month and overall TFS. In the AKD group, MELD 3.0 with albumin showed a higher iAUC value for 3-month and overall survival, but lower in 3-month and overall TFS. In the CKD group, MELD 3.0 with albumin had a higher iAUC value in 3-month survival and TFS, while showing similar values in overall survival and TFS. Lastly, in the AKD on CKD group, MELD 3.0 with albumin demonstrated a higher iAUC value for overall TFS, while showing similar values in 3-month survival and TFS, but lower in overall survival.

## 4. Discussion

As the number of patients with end-stage liver disease increases worldwide, there is an active discussion about the prognosis prediction and treatment methods for these patients. Various treatments are available, including drug therapy and interventional procedures for complications, but liver transplantation remains the ultimate treatment to date. In end-stage liver disease, liver transplantation is a highly effective and fundamental treatment method. However, the reality is that the number of liver donations available is significantly limited compared to the demand for liver transplants. Therefore, predicting the prognosis and determining the priority for liver transplantation in end-stage liver disease have always been considered of the utmost importance.

Numerous scoring systems have been developed to predict the prognosis in patients with liver cirrhosis. In particular, the MELD scoring system has played a crucial role in determining survival rates and prioritizing liver transplant candidates in patients with end-stage liver disease since its introduction. The original MELD scoring system was effectively utilized, but, over time, the need for improvements became evident to enhance its predictive accuracy. The MELD-Na score added the sodium level as a factor to the original MELD score, showing improved results. Recently, MELD 3.0, which incorporates the albumin level and a sex factor into the MELD-Na score, has been developed and is currently a subject of active research. These newly developed MELD scoring systems were initially evaluated mostly in large Western cohorts; however, recently, many Asian countries, including our research team, have been actively conducting studies on Asian cohorts as well.

In a previous study, we conducted a comparison of the original MELD, MELD-Na, and MELD 3.0 in East Asian populations. The study demonstrated that MELD 3.0 exhibited superior predictive performance compared to other scoring systems [10]. However, it was noted that the predictive performance of MELD 3.0 was reduced in East Asia compared to a Western cohort. While searching for allocation systems that could provide a more precise prognosis for patients awaiting LT in Asia, we found a newly developed scoring system known as MELD-Na-KT. This system, published in 2022, incorporates the kidney dysfunction type into the MELD-Na score [22]. Consequently, we conducted a study comparing the newly devised MELD-Na-KT scoring system with the MELD 3.0 scoring system in East Asian cohorts. Upon comparison with the recently published MELD 3.0, the MELD-Na-KT scoring system did not demonstrate superior predictive performance in the Asian population.

The main discovery from our research is the superiority of MELD 3.0 over MELD-Na-KT. Our initial hypothesis is that, in patients awaiting LT, the absolute value of serum creatinine is more crucial for the prognosis than the type of kidney dysfunction. While kidney dysfunction undoubtedly contributes to the prognosis of patients awaiting LT, the severity of renal dysfunction does not show a direct correlation with prognosis [23]. In fact, when calculating the original MELD or MELD-Na, a ceiling value of 4.0 is implemented for serum creatinine when it exceeds this threshold [24].

In previous research, Martín-Llahí and colleagues reported that the prognosis of liver cirrhosis patients can vary depending on the cause of renal failure [25]. It is worth noting that their classification of renal failure included infection, hypovolemia, HRS, and parenchymal nephropathy, which differed from the classification used in our study. There have been reports suggesting that both AKI and CKD can detrimentally affect the prognosis of liver cirrhosis patients [19,20]. However, there is limited research on which condition, either AKI or CKD, has a negative impact on the prognosis of cirrhotic patients. In particular, with the recent introduction of the concept of acute kidney disease (AKD), there has been a suggestion that AKI, AKD, and CKD may all be part of a continuum of conditions [26,27].

The second hypothesis for the superiority of MELD 3.0 over MELD-Na-KT is that albumin plays a significant role in determining the prognosis of end-stage liver disease patients. The MELD-Na-KT scoring system, derived from the MELD-Na system, does not incorporate albumin levels. Patients with end-stage liver disease typically experience a decrease in albumin levels, which appears to be highly effective in the prediction of prognosis in cirrhotic patients [28,29]. This might contribute to the relatively superior predictive performance of the MELD 3.0 scoring system.

The third hypothesis proposing the non-superiority of MELD-Na-KT over MELD 3.0 is that the classification of AKD, CKD, and related conditions may not be precise. In our study, parameters such as eGFR, the duration of renal function decline, and the presence of dialysis were used to distinguish different types of kidney dysfunction [30]. However, it is difficult to classify AKD and CKD based on the period alone. To accurately classify AKD with the potential for recovery and CKD with a lower possibility of recovery, future research on renal biomarkers appears to be necessary. If the type of kidney dysfunction can be classified more precisely, it is likely that the accuracy of MELD-Na-KT will improve in the future.

The second finding from our study is that, compared to MELD-Na-KT, MELD 3.0 exhibited higher accuracy in the female group. There have been reports suggesting that the original MELD and MELD-Na may be disadvantageous for women with relatively lower muscle mass, as they heavily rely on serum creatinine as a significant factor [31,32]. Since the introduction of the MELD scoring system, there has been a notable decrease in the likelihood of women receiving LT by 30% and waitlist mortality has increased by more than 20% [32]. To address this issue, various approaches, including serum creatinine correction, have been proposed. Specifically, the inclusion of albumin in MELD 3.0 has been effective in resolving gender-based disparities, as demonstrated in our study, exhibiting high accuracy in women.

The third key finding in our study is the disparity in the accuracy of MELD-Na-KT for the 3-month survival in the Asian population compared to the Western population (0.71 vs. 0.78). MELD-based scoring systems were initially developed in Western populations, and their accuracy has consistently been shown to be lower in East Asia [10]. This discrepancy is likely attributed to differences in liver disease etiology or racial factors. In the future, there may be a need for the development of more tailored and accurate scoring systems specifically for Asian populations.

Our study has several limitations. First, there is the difference in etiology between our study and previous Western cohorts [4]. Specifically, HBV infection is one of the prominent causes of liver cirrhosis in Korea, which differs from the United States [33]. Second, our study did not include a significant number of CKD patients undergoing dialysis; thus, it may not provide entirely accurate results.

In conclusion, our study found that among the newly developed MELD-Na-KT and MELD 3.0, MELD 3.0 demonstrated higher predictive performance in the Asian population, particularly enhancing its predictive power in female individuals. Continuous research will likely be necessary to further explore and refine liver allocation systems in East Asia in the future.

## Figures and Tables

**Table 1:** Baseline characteristics of patients at enrollment.

Variable	Total (*N* = 2080)
**Age (years)**	52.53 ± 10.95
**Sex**	
Male	1411 (67.84%)
Female	669 (32.16%)
**Etiology**	
Hepatitis B virus	820 (39.42%)
Hepatitis C virus	107 (5.14%)
Alcohol	803 (38.61%)
Fatty liver	6 (0.29%)
Autoimmune	92 (4.42%)
Others	252 (12.1%)
**Ascites**	
No	504 (24.23%)
Mild to moderate	1078 (51.83%)
Severe	498 (23.94%)
**Encephalopathy**	
No	1270 (61.06%)
Grade I–II	705 (33.89%)
Grade III–IV	105 (5.05%)
**AKD or CKD**	
Normal	1614 (77.6%)
AKD	112 (5.38%)
CKD	320 (15.35%)
AKD on CKD	34 (1.63%)
Total bilirubin (mg/dL)	7.45 ± 9.84
Serum albumin (mg/dL)	3.06 ± 0.61
Prothrombin time (INR)	1.76 ± 1.09
Serum creatinine (mg/dL)	1.23 ± 1.35
Serum Na (mEq/dL)	134.94 ± 11.69
Corrected_mGFR	79.41 ± 27.34
Original MELD	18.70 ± 9.65
MELD 3.0 with albumin	20.15 ± 9.66
MELD-Na-KT	19.69 ± 10.09

Data are reported as means ± standard deviations or medians [interquartile ranges] for continuous variables and frequencies (%s) for categorical variables.

**Table 2:** Discrimination ability.

		3-Month Survival			Overall Survival			3-Month Transplant-Free Survival			Overall Transplant-Free Survival	
All	c-index (se)	iAUC	*p*-Value *	c-index (se)	iAUC	*p*-Value *	c-index (se)	iAUC	*p*-Value *	c-index (se)	iAUC	*p*-Value *
MELD 3.0 with albumin	0.7139 (0.0183)	0.6917	0.026	0.5449 (0.011)	0.5003	0.012	0.7873 (0.0086)	0.7683	0.05	0.7219 (0.0072)	0.6713	0.037
MELD-Na-KT	0.7077 (0.0183)	0.6747		0.5405 (0.011)	0.5062		0.7845 (0.0086)	0.7573		0.719 (0.0072)	0.6656	
**Male**												
MELD 3.0 with albumin	0.6748 (0.0334)	0.6572	0.2210	0.5243 (0.0206)	0.5271	0.01	0.8058 (0.0137)	0.7808	0.0493	0.7404 (0.0123)	0.6771	0.0023
MELD-Na-KT	0.6705 (0.0334)	0.6430		0.5162 (0.0207)	0.5344		0.8019 (0.0138)	0.7753		0.7345 (0.0123)	0.6757	
**Female**												
MELD 3.0 with albumin	0.7333 (0.0216)	0.7091	0.0012	0.5578 (0.0130)	0.5154	0.002	0.7795 (0.0107)	0.7626	0.0147	0.7154 (0.0087)	0.6702	0.060
MELD-Na-KT	0.7232 (0.0220)	0.6876		0.5522 (0.0130)	0.5071		0.7759 (0.0108)	0.7487		0.7133 (0.0088)	0.6610	

Abbreviations: iAUC, incremental area under the curve; se, standard error. *: *p*-value for the c-index.

**Table 3:** Discrimination ability by kidney disfunction type.

		3-Month Survival			Overall Survival			3-Month Transplant-Free Survival			Overall Transplant-Free Survival	
	c-index (se)	iAUC	*p*-Value *	c-index (se)	iAUC	*p*-Value *	c-index (se)	iAUC	*p*-Value *	c-index (se)	iAUC	*p*-Value *
**Normal** **(*N* = 1614)**												
MELD 3.0 with albumin	0.720 (0.024)	0.713	0.001	0.552 (0.0126)	0.515	0.060	0.785 (0.0106)	0.779	0.831	0.713 (0.0084)	0.675	0.971
MELD-Na-KT	0.7089 (0.024)	0.693		0.549 (0.0125)	0.508		0.787 (0.0106)	0.780		0.715 (0.0084)	0.679	
**AKD** **(*N* = 112)**												
MELD 3.0 with albumin	0.679 (0.0496)	0.658	0.023	0.570 (0.0402)	0.533	0.151	0.765 (0.03)	0.762	0.901	0.740 (0.0289)	0.662	0.994
MELD-Na-KT	0.663 (0.0473)	0.644		0.563 (0.0393)	0.524		0.801 (0.0286)	0.784		0.753 (0.0277)	0.689	
**CKD** **(*N* = 320)**												
MELD 3.0 with albumin	0.624 (0.0434)	0.615	0.020	0.459 (0.0331)	0.608	0.110	0.747 (0.018)	0.733	0.007	0.713 (0.0166)	0.650	0.004
MELD-Na-KT	0.612 (0.0427)	0.604		0.453 (0.0325)	0.610		0.740 (0.0188)	0.726		0.707 (0.0173)	0.643	
**AKD on CKD** **(*N* = 34)**												
MELD 3.0 with albumin	0.497 (0.101)	0.517	0.09	0.408 (0.061)	0.625	0.006	0.720 (0.074)	0.710	0.601	0.627 (0.059)	0.597	0.180
MELD-Na-KT	0.473 (0.095)	0.515		0.381 (0.059)	0.646		0.723 (0.078)	0.707		0.617 (0.063)	0.566	

Abbreviations: iAUC, incremental area under the curve; se, standard error. *: *p*-value for the c-index.

## Data Availability

The datasets generated during and/or analyzed during the current study are available from the corresponding author on reasonable request.
